# Plasma activated water as resistance inducer against bacterial leaf spot of tomato

**DOI:** 10.1371/journal.pone.0217788

**Published:** 2019-05-31

**Authors:** Set Madian Perez, Enrico Biondi, Romolo Laurita, Mariarita Proto, Fabio Sarti, Matteo Gherardi, Assunta Bertaccini, Vittorio Colombo

**Affiliations:** 1 Department of Agricultural and Food Sciences (DISTAL), Plant Pathology, *Alma Mater Studiorum*—University of Bologna, Bologna, Italy; 2 Instituto de Ciencias Agronómicas y Veterinarias, Universidad de O’Higgins, Rancagua, Chile; 3 Department of Industrial Engineering (DIN), *Alma Mater Studiorum*—University of Bologna, Bologna, Italy; 4 Interdepartmental Center for Industrial Research Advanced Mechanical Engineering Applications and Materials Technology, *Alma Mater Studiorum*—University of Bologna, Bologna, Italy; 5 Interdepartmental Centre for Industrial Research Agrifood, *Alma Mater Studiorum*-Università di Bologna, Cesena, Italy; Fujian Agriculture and Forestry University, CHINA

## Abstract

Plant bacterial diseases are routinely managed with scheduled treatments based on heavy metal compounds or on antibiotics; to reduce the negative environmental impact due to the use of such chemical compounds, as pollution or selection of antibiotic resistant pathogens, the integrated control management is required. In the frame of a sustainable agriculture the use of bacterial antagonists, biological agents, plant defence response elicitors or resistant host plant genotypes are the most effective approaches. In this work, cold atmospheric pressure plasma (CAP) was applied to sterile distilled water, inducing the production of a hydrogen peroxide, nitrite and nitrate, and a pH reduction. In particular, an atmospheric pressure dielectric barrier discharge (DBD) has been used to produce plasma activated water (PAW), that was firstly assayed in *in vitro* experiments and then *in planta* through application at the root apparatus of tomato plants, against *Xanthomonas vesicatoria* (Xv), the etiological agent of bacterial leaf spot. Moreover, the transcription abundance of five genes related to the plant defense was investigated in response to PAW treatment.

PAW did not show direct antimicrobial activity against Xv in *in vitro* experiments, but it enhanced the tomato plants defenses. It was effective in reducing the disease severity by giving relative protections of *ca*. 61, 51 and 38% when applied 1 h, 24 h and 6 days before the experimental inoculation, respectively. In addition, the experiments highlighted the *pal* gene involvement in response to the PAW treatments and against the pathogen; its transcription levels resulted significantly high from 1 to 48 h until their decrease 192 h after PAW application.

## Introduction

Bacterial leaf spot of tomato caused by *Xanthomonas vesicatoria* (Xv), is widely spread in all the areas where tomato is cultivated [1,2]. The lack of effective pesticides or resistant tomato cultivars for the management of this bacterial disease, stimulates the efforts to develop sustainable strategies in the framework of the integrated control management strategies [3,4]. The control programmes for bacterial diseases of tomato plants are mainly based on prophylaxis trough diagnostic analysis carried out on seeds, to detect the pathogen presence (latent infections) [5]. Moreover, a certain degree of seed sanitation is achieved through the application of fermentation methods during the seed extraction from tomatoes and/or through physical disinfection procedures (*i*.*e*. heat treatments) directly applied to the seeds batches [6,7]. In glasshouse and open field, preventive treatments applied to the plants are based on copper compounds, antibiotics (where it is allowed) and antagonistic bacteria, that are able to reduce the pathogen population and avoid its penetration inside the plant. However, microbial resistance to copper/antibiotics poses a threat to the continued successful use of copper/antibiotics sprays for disease control [8–10]. Therefore, alternative sustainable control methods, as the use of nanoparticles or natural compounds directly active against Xv were studied and employed to control the infections [11–15]. Moreover, since the end of the nineties, the efficacy and the mechanism of action of resistance inducer molecules, acting against the pathogens through responses mediated by the host, were investigated as an alternative to the use of copper/antibiotics compounds [16]. Among all known resistance inducers, those based on the active principle acibenzolar-S-methyl (ASM), a benzo-thiadiazole (BTH) derivative, were found effective against bacterial plant diseases by strengthening the physical barriers, the pre-infectional defenses (*e*.*g*. lignins and callose production), and by producing several compounds, directly active against the pathogens (post-infectional defenses). ASM, in fact, mimics the role of salycilic acid (SA), the natural plant activator of systemic acquired resistance (SAR), which culminates in the expression of pathogenesis related proteins (PRs), in particular the PR1a is also kept as SAR marker [17]; through synergistic cross-talking pathways, ASM is also able to elicit the productions of PRs and compounds, dependant to the jasmonic acid/ethylene (JA/ET) pathway, which brings to induced systemic resistance (ISR) [18].

Those two principal defense pathways, following an oxidative burst through the production of reactive oxygen and nitrogen species (RONS), representing the earliest responses of plants to pathogen inoculation and leading to the hypersensitive response (HR) [19,20], are usually activated by the plants depending on pathogen lifestyle: in particular, host plants basically activate SA- or JA/ET-pathways against biotrophic and necrotrophic pathogens, respectively. Moreover, both defenses, dependent on SA- and JA/ET-pathways, appear to provide resistance to pathogens with mixed lifestyles (hemibiotrophs) [21,22]; however, the resistance to some pathogens appears to be through signaling pathways that involve neither of these regulators [18].

Cold atmospheric pressure plasma (CAP) can be generated by directly applying electrical energy to gas (*e*.*g*. air or noble gases) and consists of heavy particles (positive and negative ions, atoms, free radicals and excited or non-excited molecules), electrons and UV-rays [23]. CAP can be sustained in ambient air by means of a diversity of plasma source, such as corona discharge, micro hollow cathode discharge, atmospheric pressure plasma jet, gliding arc discharge, one atmospheric uniform glow discharge, plasma needle and dielectric barrier discharge (DBD) [24,25]. The biological effects of CAP treatment has been investigated in human medicine and more recently in agriculture [23,26–28]. During the last decade, different studies highlighted the direct effectiveness of cold plasma treatments in increasing the seed germination and the growth rate of various plant species, and in strengthening plant defences against bacterial pathogens [29–33]. CAP treatment of sterile deionized water (SDW) was shown to produce plasma activated water (PAW) characterized by increased acidity and conductivity, and an increased concentration of nitrates, nitrites and RONS, in particular hydrogen peroxide [28,34,35]. The antimicrobial activity of PAW against bacterial and fungal human pathogens was highlighted in *in vitro* experiments [28]. In addition, the positive effect of PAW on tomato seeds germination time and rate has been demonstrated [36].

This study was aimed to assess the *in vitro* antimicrobial activity of PAW against Xv, and its ability to reduce the bacterial leaf spot disease severity by eliciting induced resistance in *in planta* experiments. Furthermore, transcriptomic analyses were performed in tomato plants treated with PAW to define the transcription kinetics of selected genes regulating pre- and post-infectional plant defense responses as phenylalanine ammonia-lyase (*pal*), ethylene-response factor (*erf1*), pathogenesis related protein 1 (*pr1a*; SAR marker), endochitinase (*pr4*; ISR marker) and thaumatin-like protein (*pr5*) genes [18,19,22,37–39].

## Materials and methods

### PAW production

**Fig 1:**
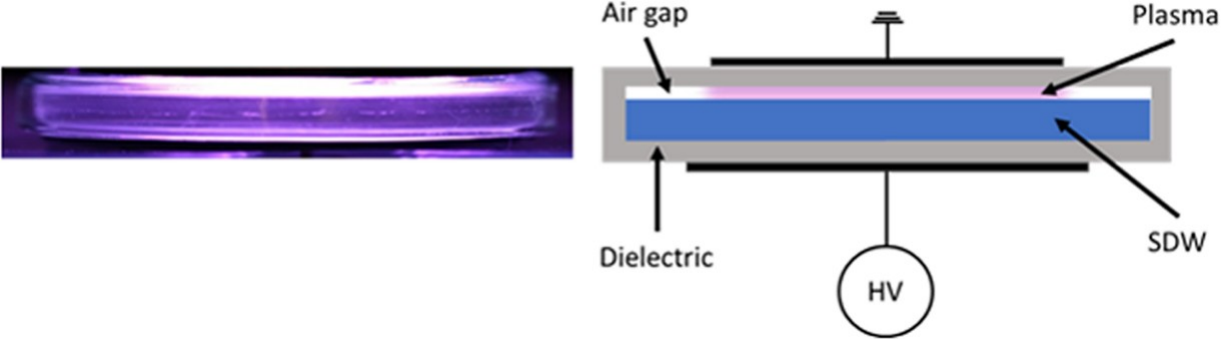
Water activation through plasma treatment. Picture (left) and schematic (right) of cold atmospheric pressure plasma (CAP) treatment of sterile distilled water (SDW) by means of nanosecond pulsed DBD for PAW production.

PAW was produced following the methodology described by Laurita *et al*. [28]. Briefly, CAP was generated by a DBD reactor consisting of a polystyrene cylindrical case (thickness 2 mm, diameter 94 mm) acting both as a liquid container and dielectric barrier (Fig 1). Aliquots of 80 ml of SDW were treated in a closed environment without recirculation; the volume of the reactor was filled with ambient air, which was used as plasma gas. Two circular aluminum foils acted as electrodes: the liquid-side electrode (diameter 89 mm) was connected to a nanosecond pulsed high voltage generator, while the gas-side electrode (diameter 80 mm) was grounded. The plasma source was driven by the HV generator producing pulses with a slow rate of few kV/ns and 50 mJ of energy per pulse (FID GmbH–FPG 20-1NMK).

PAW was obtained operating for 10 min the source at a peak voltage of 20 kV, with a pulse repetition frequency of 1 kHz and an air gap of 1 mm and it was applied both in *in vitro* and *in planta* experiments between 1 h and 3 h after CAP treatment.

### Detection of hydrogen peroxides (H_2_O_2_), nitrates (NO_3_^-^) and nitrites (NO_2_^-^) in PAW.

The pH and concentrations of H_2_O_2_, NO_3_^-^ and NO_2_^-^ induced by plasma treatment were measured using the Amplex Red Hydrogen Peroxide Assay Kit (Thermo Fisher Scientific, Waltham, MA, USA) and Nitrate/Nitrite Colorimetric Assay (Roche, Basel, Switzerland) according to the manufacturer’s protocol. PAW was diluted 100-fold in PBS to avoid any influence of pH on the measurement. A microplate reader (Rayto, Shenzhen, P.R. China) was used to measure solutions absorbances. Measurements were repeated three times.

### Bacterial strains

The Xv strain IPV-BO 2684, isolated from symptomatic tomato plants in Italy and stored in the Phytobacteriology collection of the University of Bologna, was grown on the nutritive medium GYCA (glucose, yeast, calcium carbonate agar) [40] for 48 h at 27°C.

### *In vitro* experiments

The *in vitro* antimicrobial activity of PAW was tested with the diffusion and broth dilution methods recommended by the Clinical and Laboratory Standards Institute [41] and slightly modified, by using LB medium instead of Mueller-Hinton substrate.

#### Diffusion method

LB agar plates were contaminated with 200 μL of bacterial water suspension calibrated at the spectrophotometer (OD = 0.01_600nm_; approx. 10^7^ CFU/mL). A drop (60 μL) of each treatment was deposited onto an antibiogram disc (diameter 0.9 cm), previously kept in the middle of each contaminated Petri dish; the plates were then incubated for 24 h at 27°C. The antibacterial effect, shown as an inhibition halo was then measured (mm) by subtracting the antibiogram disc diameter from the halo diameter. SDW and acibenzolar-S-methyl (ASM, 75 ppm) were used as negative controls, while streptomycin sulphate (10,000 ppm) and H_2_O_2_ (30%) as positive controls. The assays was repeated three times and the data were elaborated with ANOVA test (p 0.05) using SPSS version 15.0 for Windows.

#### Broth dilution method

Sterile 50 mL Falcon tubes containing 15 mL LB broth were contaminated with 150 μL of bacterial aqueous suspension calibrated at the spectrophotometer (OD = 0.1_600nm_; approx. 10^8^ CFU/mL). Each Falcon tube was then added with 300 μL of each treatment and incubated at 27°C for 24 h at 80 rpm in a rotative incubator. The bacterial population was evaluated at 24 h by collecting 1 mL contaminated LB broth. Each sample was 10-fold diluted and, 10 μL from each dilution were dropped onto GYCA medium; the plates were incubated at 27°C for 48–72 h and the bacterial populations were evaluated by counting the colonies. SDW and acibenzolar-S-methyl (ASM, 75 ppm) were used as negative control, while streptomycin sulphate (10,000 ppm) and H_2_O_2_ (30%) as positive controls. The assay was repeated three times and the data were elaborated with ANOVA test (p 0.05) using SPSS version 15.0 for Windows.

### *In planta* experiments

#### Efficacy of PAW against Xv in tomato plants

Under greenhouse conditions three experiments were carried out on two different tomato plant cultivars, cv. Moneymaker and VF-10 [7], disposed in randomized blocks (3 plants x 4 blocks/treatment). The tomato plants were uprooted at 3^rd^-4^th^ leaf stage and the root apparatus was soaked for 10 min into 500 mL of ASM, (75 ppm; positive control), PAW and SDW (negative control); then, the plants were put back into the pots. The treatments were carried out from 1.5 hours to 7 days before the experimental inoculation with the pathogen (BPI) as shown in Table 1.

**Table 1:** *In planta* experiments: Types and time of treatment application carried out at root *apparatus* of tomato plants (cv. Moneymaker and VF-10) under greenhouse conditions.

No. Experiment/ Treatments	Application timing (days/hours BPI^a^)
**No. 1 and 2 cv. Moneymaker and VF 10**	
Sterile distilled water (SDW)	6 d
Plasma activated water (PAW)	6 d
acibenzolar-S-methyl (ASM)	7 d
**No. 3 cv. VF-10**	
SDW	1.5 h
PAW-1	2 d
PAW-2	1 d
PAW-3	1.5 h
ASM	7 d

^a^ BPI: Before pathogen inoculation

The inoculation was carried out by spraying a water suspension (OD_600nm_ = 0.01; *ca*. 10^7^ CFU/mL), containing Xv strain IPV-BO 2684, on the leaf surfaces; tomato plants were then sealed in polyethylene (PE) bags for two days to favour the water congestion and to allow the pathogen penetration. The disease assessments were carried out by counting the leaf spots (on 5 leaves/plant) 21 days after the experimental inoculation, for a more clear and precise evaluation of the disease severity. The controlled conditions, hold until the disease assessment, were 16 h of day light and 8 h of darkness, 30°C and 24°C during day and night respectively; moreover, the relative humidity (RH) was maintained up to 70–75% according with the pathogen requirements. Data were elaborated with ANOVA test (Duncan's, p 0.05) and the relative protection (RP) of each treatment was calculated [(No. leafspots in SDW control plants—No. leafspots in treated plants)/ No. leafspots in SDW control plants].

Selected symptomatic leaf samples were used for the isolation and identification of the pathogen. The leaf surface was sterilized with 2% sodium hypochlorite. Necrotic lesions were aseptically collected and crushed into a mortar with 2 mL of sterile distilled water; 30 μL of the 10^−1^ and 10^−2^ diluted extract were dropped onto GYCA medium and incubated up to 48–72 h. Xv-like colonies were subcultured and identified with molecular assays [5,42].

### Evaluation of defense-related gene transcription

#### Treatments and sample collection

Under greenhouse conditions, tomato plants cv. Moneymaker (2 plants x 4 blocks/treatment) at 3^rd^-4^th^ leaf stage were uprooted and their root apparatus was drenched into the following treatments: SDW (negative control), PAW, ASM (75 ppm, positive control), jasmonic acid (JA, Sigma cod. J2500, 100 mM, positive control). Two further tomato plant batches, PAW and untreated ones, were inoculated with Xv by spraying a water suspension of the bacterial pathogen (*ca*. 10^7^ CFU/mL) on the leaf surfaces (PAW+Xv-I and Xv-I, respectively labelled); the inoculated tomato plants were then sealed in polyethylene (PE) bags for two days (humid chamber). Furthermore, untreated and non-inoculated (NT) tomato plants were used as negative control and calibrator for data normalisation.

The three youngest tomato leaves were collected from each replicate/treatment at six time points: 1 h (T0), 7 h (T1), 24 h (T2), 48 h (T3), 120 h (T4) and 192 h (T5). The tissues were flash frozen in liquid nitrogen and stored at -80°C until RNA extraction. The greenhouse conditions were set as 16 h light at 30°C and 8 h dark at 24°C, and the RH was maintained at 70–75% until the end of the experiment.

#### RNA extraction

Approximately 100 mg of tomato leaves stored at -80°C were ground in liquid nitrogen and homogenized in 1.2 mL of extraction buffer [guanidine iso-thyocianate, 4 M; CH_3_COO^-^Na^+^ (pH 5), 0.2 M; NaEDTA (pH 8), 0.025 M; PVP 40, 2.5%] [43]. The extract was transferred into 2 mL Eppendorf tube and centrifuged at room temperature at 17,500 *g* for 5 min; 1 mL of the supernatant was added with 100 μL of Na-lauroyl sarcosine 30%, and mixed by inversion. The sample was then incubated at 70°C for 30 min. The total volume of the extract was placed into QIA shredder spin columns, then the extraction continued with Qiagen RNeasy Plant Minikit (cat. N° 74904) in combination with RNase-Free DNase Set (Qiagen; cat. No. 79254) to digest DNA following the instructions of the manufacturer. The total RNA, eluted in 50 μL of nuclease free distilled water, was stored at -20°C. Quantity and quality (ratio A_260nm_/A_280nm_) of 3 μL of RNA extracts were evaluated using Tecan Infinite 200 Pro NanoQuant instrument and i-control software (Tecan Group Ltda., Switzerland). The quality was also assayed by loading 5 μL of RNA extracts into a 2% agarose gel; the electrophoresis was performed at 40 V for 100 min in TAE buffer. The gel was stained in ethidium bromide solution (0.03%) for 20 min and distained in distilled water for 5 min; visualisation was then carried out under UV light (312 nm). All RNA samples were diluted at 100 ng/μL before the molecular assays.

#### Reverse transcription quantitative polymerase chain reaction (RT-qPCR) analysis

Specific primers for RT-qPCR were designed on the sequences of reference and target genes by using Primer Express 2.0 software (Applied Biosystems, Foster City, CA). The primers used for RT-qPCR and the accession numbers of the gene sequences are listed in Table 2. The RT-qPCR assay was performed using ABI7000 v 1.2.3 sequence detection system. Each reaction was carried out with one-step method, in a final volume of 25 μL containing 1 U Murine Leukemia Virus (M-MLV; Promega, cod. 1705), 12.5 μL Go*Taq* qPCR Master Mix (Promega, No. A6001, SYBR Green/ROX chemistry), 0.25 μL CXR reference dye, primers at 400 nM (*β-actin*, *erf1*, *pr1a*, *pr4* and *pr5* and 100 nM (*pal*) (Invitrogen, cod. A9267) and 1 μL of RNA template (100 ng/μL). The primers specificity was evaluated on RNA extracts by dissociation curves analysis, in order to exclude non-specific amplifications or primer dimers presence. The thermal profile was set up as follows: 30 min at 48°C, 95°C for 10 min, followed by 40 cycles of 95°C for 10 s, and 60°C for 1 min. Dissociation curves were performed at 95°C for 15 s, 60°C for 20 s and 95°C for 15 s. Data were analyzed using SDS1.2 software (Applied Biosystems). The efficiency of each primer pair was determined using RNA 10-fold dilution series (calibration curves) to determine the fold changes. Exponential amplification was plotted on a logarithmic scale, and dye fluorescence as a function of cycle number (R_n_) was set to 0.32 for each RT-qPCR plate to obtain the cycle threshold (Ct) values.

**Table 2:** Primer sequences used for qRT-PCR.

Gene (Accession No.)	Primer	Sequence (5’ - 3’)	Dissociation curve (°C)	Reference
*β-actin* (KY008745)	Forward	AGCTCCTCCATTGAAAAGAACTATG	73.0	This study
	Reverse	GGTAATAACTTGTCCATCAGGCAA		
*pr1a* (KY609511)	Forward	TGTTGGTGGAAAAATGTGTGGA	74.5	This study
	Reverse	GAGTTGCGCCAGACTACTTGAGT		
*pr4* (KY609512)	Forward	TATGAACGTTAGGGCAACGTATCA	73.0	This study
	Reverse	CAGTTTATGTTTTGCGGATTGTACA		
*pr5* (KY609513)	Forward	CCAGTTTAGCAACCTAGATTTCTGG	74.0	This study
	Reverse	TTAAATCCATCGACTAAAGAAATGTCC		
*pal* (KY614301)	Forward	TCAGCACTTTGGACATGGTTAGTC	75.0	This study
	Reverse	AGAACTTCAATTCCTTGCAAATCC		
*erf1* (KY614303)	Forward	AACTCAATGGCTAGGGCTTGTTT	75.0	This study
	Reverse	TTTGCTATTTTCTGTCCACTTCAAAG		

All gene transcription levels were reported as Mean Normalised Transcription relative to *β-actin*, used as the reference gene [44,45]. Gene transcription was determined by the 2^−ΔΔCT^ method adjusted by amplification efficiency for each trancripts [46]. The mean data obtained were analysed by ANOVA (p 0.05) followed by Student-Newman-Keuls (SNK) multiple range test.

## Results and discussion

The plasma treatment of SDW induced the production of NO_2_^-^, NO_3_^-^ and H_2_O_2_. As reported by Laurita *et al*. [28], NO_2_^-^ completely disappeared few minutes after the plasma treatment, due to its reaction with H_2_O_2_ in acidic liquids. One hour after treatment, H_2_O_2_ and NO_3_^-^concentrations (approx. 20 mg/L and 120 mg/L, respectively) in PAW resulted stable for at least 2 hours at room temperature (Table 3); so the delay time between the production and the PAW treatment of plants was mantained between 1 h and 3 h in order to have stable concentrations of H_2_O_2_ and NO_3_^-^.

**Table 3:** pH, hydrogen peroxide and nitrate nitrite concentrations in SDW exposed for 10 min to nanosecond pulsed DBD, 1 and 3 h after treatment.

	pH	Hydrogen peroxide [mg/l]	Nitrates [mg/l]
**Untreated solution**	5,5±0,1	0	0
**1 h delay time**	2,78±0,47	22±0,24	121,4±25,9
**3 h delay time**	2,78±0,47	21,1±0,47	126±2,3

### Antimicrobial activity

PAW did not show antimicrobial activity against Xv in the *in vitro* experiments using the diffusion method; the results were comparable to those of SDW and ASM (negative controls). Meanwhile, the positive controls (streptomycin and H_2_O_2_) inhibited the pathogen growth causing a mean inhibition haloes of 3.0 cm and 3.8 cm, respectively (S1 Fig).

The lack of PAW inhibition activity against Xv was confirmed in the experiments carried out using the broth dilution method. 24 h after contamination, the bacterial population in the Falcon tubes added with PAW rose up to approx. 10^9^ CFU/mL, as occurred in the negative controls (SDW and ASM); on the contrary, streptomycin sulphate and H_2_O_2_ completely killed the strain IPV-BO 2684 (S2 Fig). In a recent work [28], in which the efficacy of PAW against *Candida albicans* and *Staphylococcus aureus* was studied using the time-kill method [47], it was pointed out that the loss of PAW antimicrobial activity was related to the post discharge chemistry: 16 minutes after the treatment, the concentration of H_2_O_2_ resulted 100 μM (*ca*. 0.0003%), whereas NO_2_^-^ was not detectable by the instruments, and PAW did not show any efficacy against the two pathogens. The lack of antimicrobial activity was related to the absence of peroxynitrous acid (ONOOH), NO_2_^-^ and to the low concentration of H_2_O_2_. In the PAW used for the *in vitro* experiments of the present study, ONOOH and NO_2_^-^ were not detected, and H_2_O_2_ concentration resulted low as well [28], since the experiments were carried out more than 1 h after plasma treatment: this partially explains the lack of PAW efficacy against Xv. In addition, the assays used in the *in vitro* experiments of this study were based on diffusion and broth dilution methods, as suggested by Clinical and Laboratory Standards Institute (CLSI, formerly NCCLS) [41]; these methods differed from those proposed and used in Laurita *et al*. (2015) [28] and in the other studies in which PAW was employed [48,49]. In those studies, it was taken into account not just the time elapsed between the PAW production and its use in the *in vitro* experiments, but also the effect of exposure time of the bacterial suspension to higher volumes of PAW: Laurita *et al*. [28] added 10 mL of PAW to 100 μL of pathogen suspension and evaluated the decrease of pathogen concentration at different time points after the treatment (time-kill method).

The presence of H_2_O_2_ and NO_3_^-^, and the low pH suggested the possible use of PAW in controlling plant pathogens, as activator of a defence mechanisms mediated by the plant host. The reactive oxygen species (ROS), such as ∙O_2_, ∙OH and H_2_O_2_, are known to be one of the upstream responses of the plant during a pathogen attack [19,50–54].

The *in planta* experiments conducted on tomato plants were therefore aimed to evaluate the indirect effect of PAW (applied at the roots) against Xv (experimentally inoculated at the leaves), through the host-mediated induced resistance.

### Induced resistance *in planta*

In all the experiments carried out *in planta* no phytotoxicity or reduction in the vegetative growth was observed in PAW treated non-inoculated plants (used as control).

**Fig 2:**
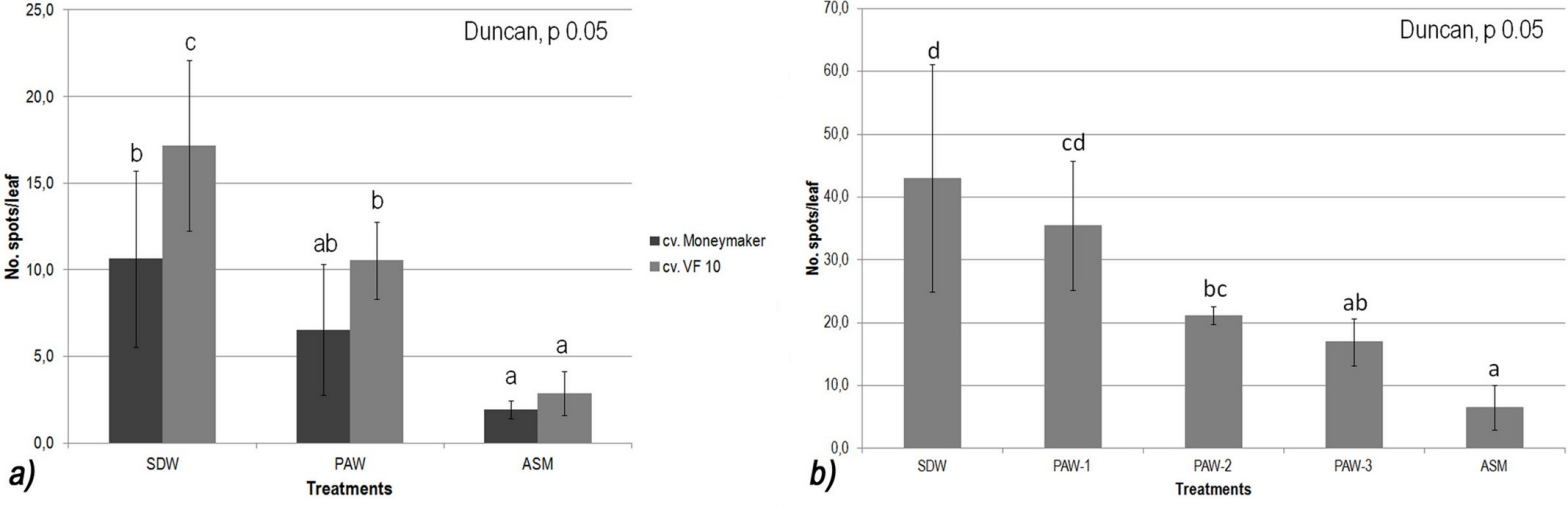
*In planta* experiments on two different tomato plant cultivars. a) Results of the *in planta* efficacy of plasma activated water applied 6 days before pathogen inoculation at root apparatus (PAW-R), compared to the negative (SDW-R) and positive controls (ASM-R), against bacterial leaf spot caused by *Xanthomonas vesicatoria* (strain IPV-BO 2684) on tomato plants cv. Moneymaker (dark grey histogram) and cv. VF 10 (grey histogram) grown under greenhouse conditions. b) Results of the *in planta* efficacy of plasma activated water applied at root apparatus at different times before pathogen inoculation (BPI) (PAW-1: 2 days BPI, PAW-2: 1 day BPI, PAW-3: 1.5 hours BPI), compared to that of the negative (SDW) and positive controls (ASM), against bacterial leaf spot caused by *Xanthomonas vesicatoria* (strain IPV-BO 2684) on tomato plants cv. VF-10 grown under greenhouse conditions. Different letters denote significant differences according to the Duncan's test (p 0.05).

In the first experiment, tomato plants cv. Moneymaker treated with PAW 6 days BPI, showed a medium level of disease severity (*ca*. 6 spots/leaf) compared to the plants treated with SDW (negative control; *ca*. 11 spots/leaf) and those treated with ASM (2 spots/leaf) (Fig 2); the RP of PAW was approx. 38%.

The results of the second *in planta* experiment on tomato cv. VF-10 (Fig 2), confirmed that PAW treatment applied 6 days BPI was able to significantly reduce the number of bacterial leaf spots (11 spots/leaf), with respect of SDW (17 spots/leaf), meanwhile, ASM treated plants showed again the lowest statistical number of spots per leaf (*ca*. 3 spots/leaf). The RP provided by PAW application was approx. 38%. as occurred in the first experiment on tomato cv. Moneymaker. It was clear that the tomato plants cv. Moneymaker resulted less susceptible to Xv than those of cv. VF-10; even though, the RP among the treatments were comparable, thus confirming the efficacy of PAW in reducing the disease severity.

The third experiment, carried out on cv. VF-10 plants, was characterized by a consistently higher disease pressure with respect of the previous two experiments. The disease severity on plants treated with PAW 2 days BPI (PAW-1) resulted lower (*ca*. 35 spots/leaf) than that observed on SDW treated plants (*ca*. 43 spots/leaf), but statistically higher with respect of plants treated with PAW applied 1 day (PAW-2) and 1.5 hours (PAW-3) BPI (*ca*. 21 and 17 spots/leaf respectively). RPs provided by PAW-1, PAW-2 and PAW-3 were *ca*. 18%, 51% and 61% respectively; ASM treated tomato plants showed the statistical lowest disease severity (*ca*. 6 spots/leaf) among all treated plants (Fig 2), giving the highest levels of RPs which ranged approx. 82% to 85% in the three experiments as expected [12,16,17]. These results confirmed the efficacy of PAW as possible resistance inducer against bacterial diseases of tomato plants.

The time elapsed between PAW application and pathogen inoculation played an important role in the plant defences induction. In all *in planta* experiments, it was observed a reduction of disease severity in the treatments with PAW; in particular, the highest RP was provided by PAW applied 1.5 h or 1 day BPI. The results suggested that the host mediated response was triggered by the PAW treatment and the induced disease reduction was related to the time elapsed between the treatment application and the pathogen inoculation. The ROS and/or RNS, induced by CAP treatment in SDW, might be linked to signalling pathways in tomato that establish a correlation between the disease reduction and the activation of defence mechanisms, and that were investigated with the transcriptomic analysis on genes related to the main induced resistance pathways [19].

### Gene transcription

Five genes (*pal*, *erf1*, *pr1a*, *pr4* and *pr5*), regulating pre- and post-infectional plant defence responses [18,19,22,37–39] were assayed by RT-qPCR on cv. Moneymaker, to evaluate their transcription abundance in leaves after the root application of different treatments and/or inoculation with the pathogen. The *β*-*actin* resulted a reliable reference gene, because its abundance in PAW treated plants, and in the negative (NT and SDW) and positive (ASM and JA) control plants were equivalent. The use of the negative control treatment (SDW), whose type of application was similar to that of the other treatments (PAW, ASM and JA), allowed to measure the intensity of the plant defence response: the application method, in fact, could have brought side stress effects dependant to the wounds produced during the treatment application [39].

#### *pal* gene

**Fig 3:**
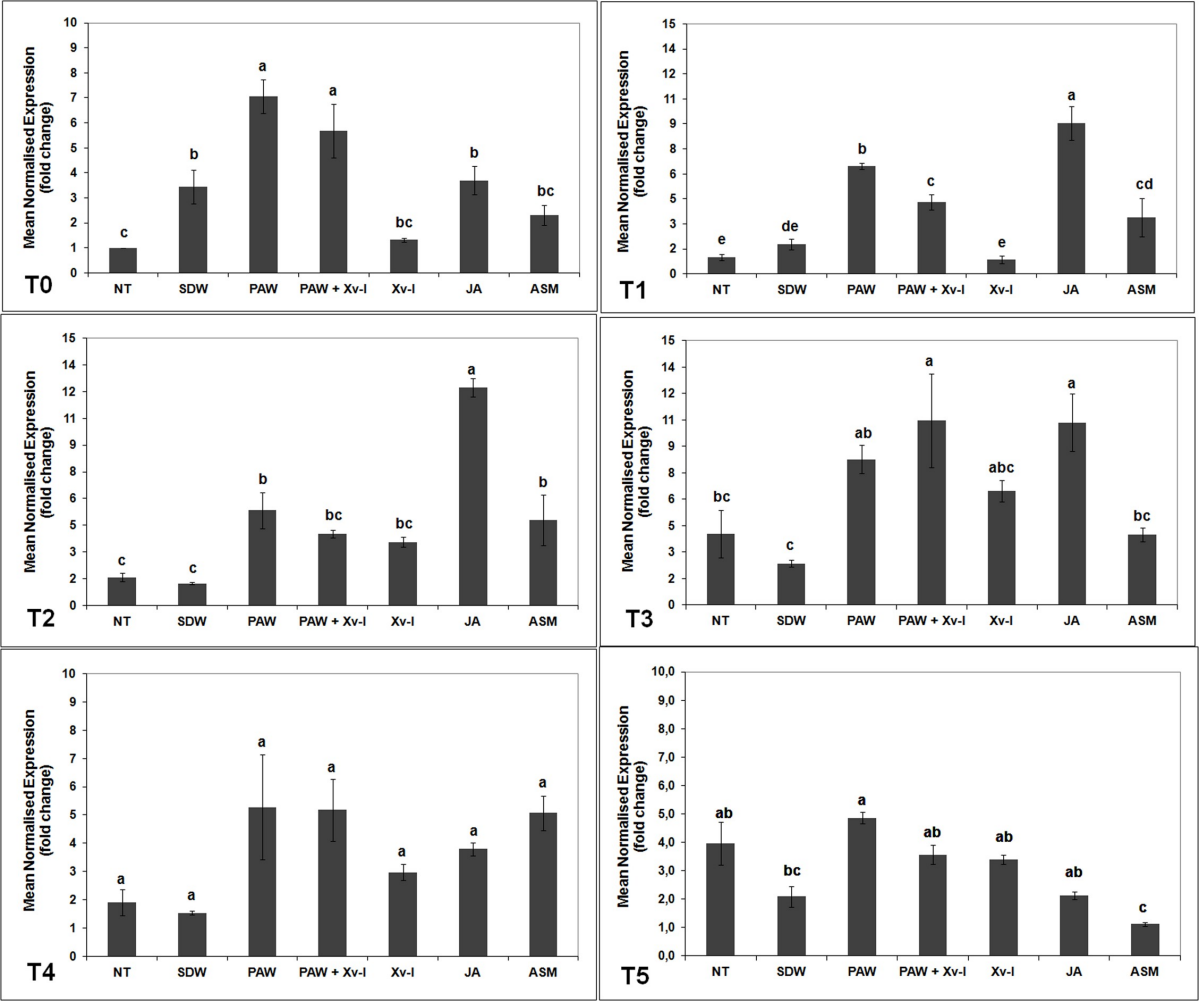
*pal* transcription kinetics in tomato leaves. The graph shows the *pal* induction at each time point (T0: 1 h; T1: 7 h; T2: 24 h; T3: 48 h; T4: 120 h; T5: 192 h) after treatment/inoculation and the standard error (±SE). Different letters denote significant differences according to the SNK test (p 0.05). NT: non-treated/non-inoculated; SDW: treated plants at root *apparatus* with sterile distilled water; PAW: treated plants at root *apparatus* with plasma activated water; PAW+Xv-I: treated plants at root *apparatus* with plasma activated water and inoculated with *Xanthomonas vesicatoria* sprayed at leaves; Xv-I: inoculated with *Xanthomonas vesicatoria* sprayed at leaves; JA: treated plants at root *apparatus* with jasmonic acid; ASM: treated plants at root *apparatus* with acibenzolar-S-methyl.

One hour after treatment (T0), the number of *pal* gene transcripts significantly increased in tomato plants treated with PAW and in those treated with PAW and inoculated with the pathogen (PAW+Xv-I) by *ca*. 7.0- and 7.5-fold, respectively, when compared to untreated and non-inoculated plants (NT, *ca*. 1-fold), to those pre-treated with SDW (3.4-fold) and to those inoculated with the pathogen (Xv-I, 1.3-fold). The plants treated with JA (positive control) showed a statistical increase of *pal* gene transcripts, compared to NT, SDW and Xv-I ones; nonetheless, such increase resulted statistically lower than that observed in plants treated with PAW (Fig 3). After 7 h (T1), the *pal* gene transcripts increase evaluated in the treatments PAW and PAW+Xv-I resulted lower (*ca*. 6.5- and 4.4-fold, respectively) compared to those observed at T0, since it resulted significantly higher than that found in NT, SDW and Xv-I (*ca*. 1-, 1.8- and 0.9-fold, respectively). In the treatment ASM (positive control), a higher transcript abundance was present compared to that of negative controls (NT and SDW) and of Xv-I, but lower than that resulted in PAW, PAW+Xv-I and JA; in particular, the plants treated with JA showed the highest increase of transcription activity (*ca*. 9.0-fold, Fig 3).

Twentyfour hours after treatment/inoculation (T2), the *pal* gene transcription activity in the treatments PAW and PAW+Xv-I resulted still higher (*ca*. 5.3- and 4.0-fold) than that in the control treatments NT and SDW (*ca*. 1.6- and 1.2-fold), while it was comparable to that observed in Xv-I (*ca*. 3.6-fold). The tomato plants treated with ASM produced *pal* gene transcriptional activity of *ca*. 4.8-fold, higher than that evaluated in NT and SDW; plants treated with JA showed the highest transcripts increase up to *ca*. 12.2-fold (Fig 3). After 48 h (T3), a general increase of the *pal* transcripts was recorded in all treatments, but it was still possible to evaluate statistical differences among them: in PAW and PAW+Xv-I tomato plants, the number of transcripts significantly increased (*ca*. 8.3- and 10.5-fold) with respect of those in NT and SDW (*ca*. 4.0- and 2.3-fold), whose transcription of *pal* resulted similar to that of plants treated with ASM (*ca*. 4.0-fold). On the contrary, plants treated with JA showed high number of transcripts (*ca*. 10.4-fold), comparable to that observed in the treatment PAW+Xv-I (Fig 3, T3). After 120 and 192 h (T4 and T5, respectively), the transcriptional level of *pal* gene decreased in all treatments: however at T4 the transcription resulted apparently higher in PAW, PAW+Xv-I, Xv-I, JA and ASM (*ca*. 5.3-, 5.2-, 3.0-, 3.8- and 5.0-fold), in comparison to that of NT and SDW (*ca*. 1.9- and 1.5-fold, respectively), but the differences were not significant. Finally at T5, the *pal* gene transcription in PAW decreased down to *ca*. 4.9-fold, still being higher in comparison to that found in the treatments NT, SDW, PAW+Xv-I, Xv-I, JA and ASM (*ca*. 4.0-, 2.1-, 3.6-, 3.4-, 2.2- and 1.1-fold) (Fig 3).

The rapid induction of *pal* gene transcription 1 hour after PAW treatment might be explained by the PAW chemical composition, characterised by low pH and the presence of H_2_O_2,_ inducing the plant hypersensitive response (HR), and also acting as signal to induce local and systemic resistance [54–60]. The combination of these chemical characteristics of PAW could allow a similar, and in few cases, larger increase of *pal* gene transcription in comparison with positive controls (ASM and JA).

In Fig 3, it can also be observed the effect of the treatment application method (explantation, root drenching and transplant): the tomato plants treated with SDW (negative control) showed an increase of *pal* gene transcripts after 1 hour, significantly higher in comparison to NT and Xv-I plants, even though such increase resulted lower of that of PAW treatments.

Moreover, the tomato plants responded against the pathogen (Xv-I) 24 h (T2) after the experimental inoculation, and they increased the *pal* gene transcription up to *ca*. 3.6-fold, higher than that evaluated in negative control treatments (NT and SDW). The increase of *pal* gene transcription was evaluated until 48 h (T3) as almost double in comparison to that observed at 24 h thus confirming that the plants started at a late time to activate these resistance defences. On the other hand, at 48 h the plants treated with PAW in combination with pathogen inoculation (PAW+Xv-I) resulted the highest in the *pal* gene transcript abundance, comparable to those of JA treatment. This was due to the response of the tomato plants to the infection plus the response to PAW treatment. Interestingly, the RP on plants treated with PAW 48 h before the inoculation with the pathogen resulted *ca*. 18%. Still, the results highlighted the *pal* involvement in response to PAW treatments and against the pathogen.

#### *erf1* gene

**Fig 4:**
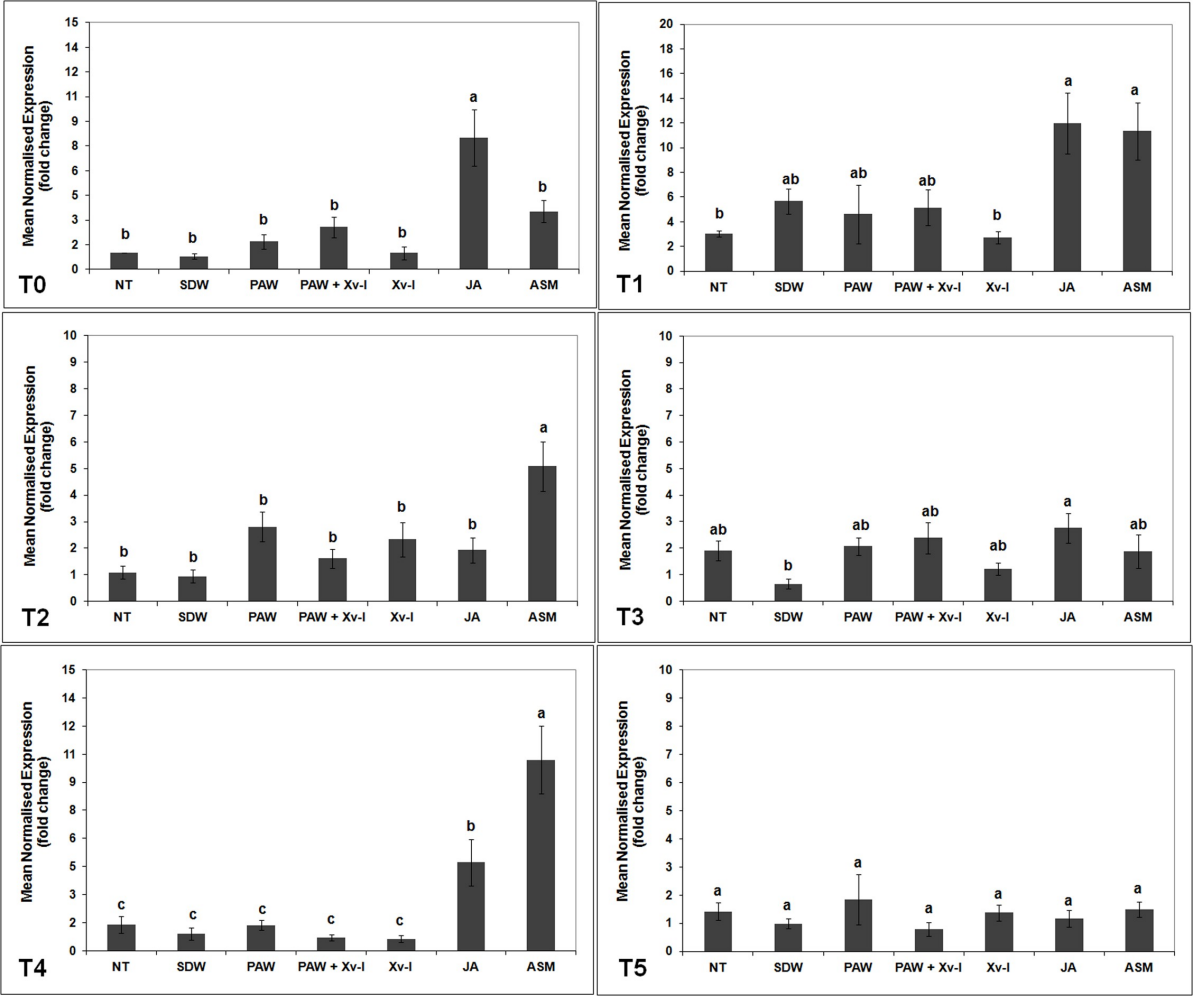
*erf1* transcription kinetics in tomato leaves. The graph shows the *erf1* induction at each time point (T0: 1 h; T1: 7 h; T2: 24 h; T3: 48 h; T4: 120 h; T5: 192 h) after treatment/inoculation and the standard error (±SE). Different letters denote significant differences according to the SNK test (p 0.05). NT: non-treated/non-inoculated; SDW: treated plants at root *apparatus* with sterile distilled water; PAW: treated plants at root *apparatus* with plasma activated water; PAW+Xv-I: treated plants at root *apparatus* with plasma activated water and inoculated with *Xanthomonas vesicatoria* sprayed at leaves; Xv-I: inoculated with *Xanthomonas vesicatoria* sprayed at leaves; JA: treated plants at root *apparatus* with jasmonic acid; ASM: treated plants at root *apparatus* with acibenzolar-S-methyl.

In the plants belonging to PAW and PAW+Xv-I treatments, it was not shown a significant increase in the abundance of *erf1* gene transcriptional activity from T0 to T5 in comparison to the negative controls (NT and SDW). Seven h (T1) after treatment, a low increase of *erf1* gene transcription was observed in PAW, PAW+Xv-I and SDW (*ca*. 5.2-, 4.6- and 5.7-fold, respectively), when compared to that of NT and Xv-I (*ca*. 3.0- and 2.7-fold, respectively) (Fig 4), very likely due to the stress occurred during the treatment application [39]. Even the inoculation with the pathogen (Xv-I) did not elicit the *erf1* gene transcription in none of the time points (from T0 to T5), suggesting that pathogen infection did not involve the ethylene pathway as first response. On the contrary, in the tomato plants treated with JA, the abundance of *erf1* transcripts increased since the beginning of the experiment. At 1 h (T0), the transcription resulted approx. 8.0-fold, and after 7 h (T2) it reached 12.0-fold, significantly higher than in the plants of negative control treatments (NT and SDW) and in those inoculated with the strain IPV-BO 2684 (Xv-I) at both time points, thus demonstrating the activation of JA/ethylene pathway which usually brings to ISR [22,61,62]. The transcriptional level of *erf1* gene in plants treated with ASM resulted statistically higher than that of negative control treatments at 7 (T1), 24 (T2) and 120 h (T4) (*ca*. 11.3-, 5.1- and 10.2-fold, respectively) (Fig 4). The delay of *erf1* gene transcription in plants treated with ASM, with respect of those treated with JA active since T0, confirmed the presence of inter-signaling pathways between the salicylic acid (SA) and JA/ethylene pathways [22,63].

#### *pr1a*, *pr4*, and *pr5* genes

**Fig 5:**
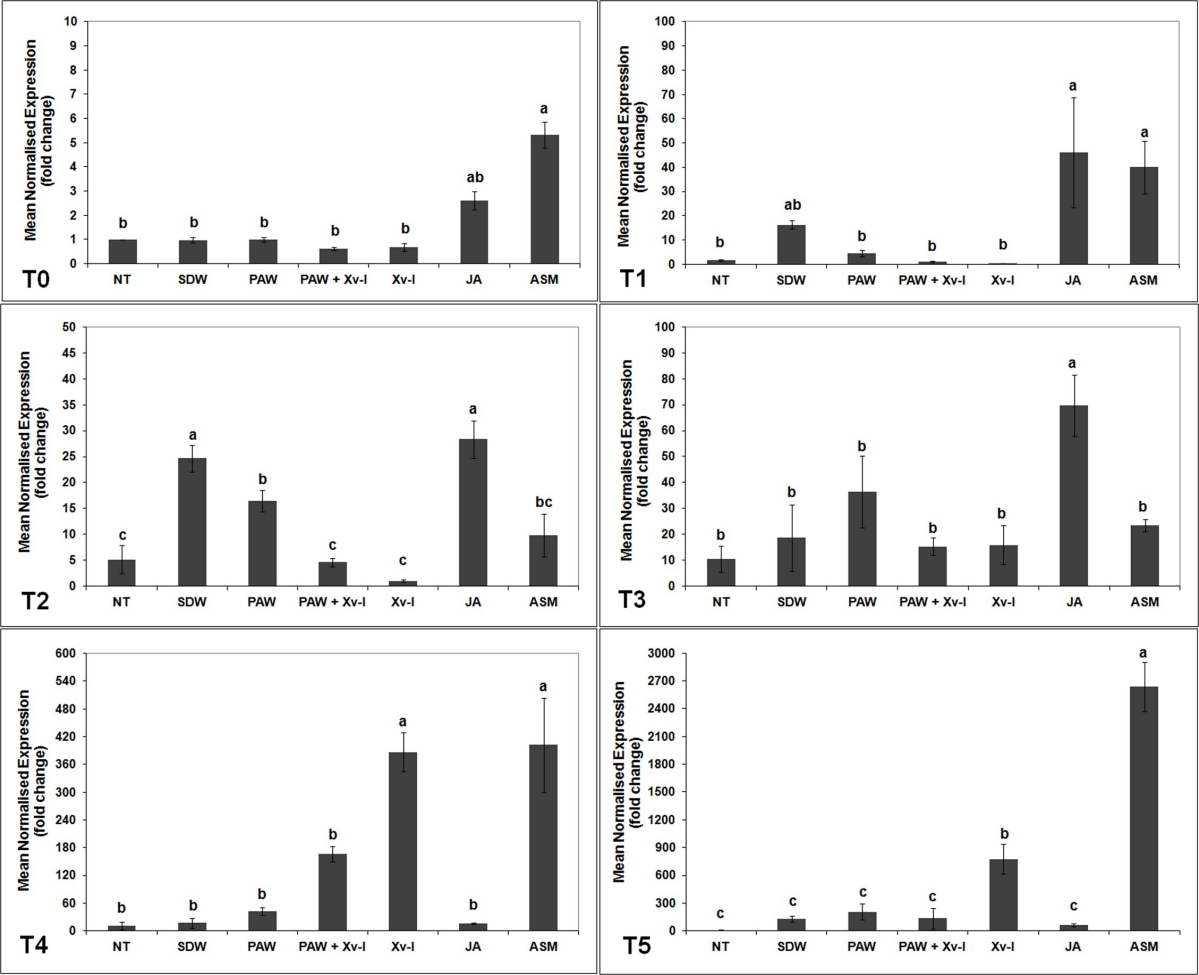
*pr 1a* transcription kinetics in tomato leaves. The graph shows the *pr 1a* induction at each time point (T0: 1 h; T1: 7 h; T2: 24 h; T3: 48 h; T4: 120 h; T5: 192 h) after treatment/inoculation and the standard error (±SE). Different letters denote significant differences according to the SNK test (p 0.05). NT: non-treated/non-inoculated; SDW: treated plants at root *apparatus* with sterile distilled water; PAW: treated plants at root *apparatus* with plasma activated water; PAW+Xv-I: treated plants at root *apparatus* with plasma activated water and inoculated with *Xanthomonas vesicatoria* sprayed at leaves; Xv-I: inoculated with *Xanthomonas vesicatoria* sprayed at leaves; JA: treated plants at root *apparatus* with jasmonic acid; ASM: treated plants at root *apparatus* with acibenzolar-S-methyl.

**Fig 6:**
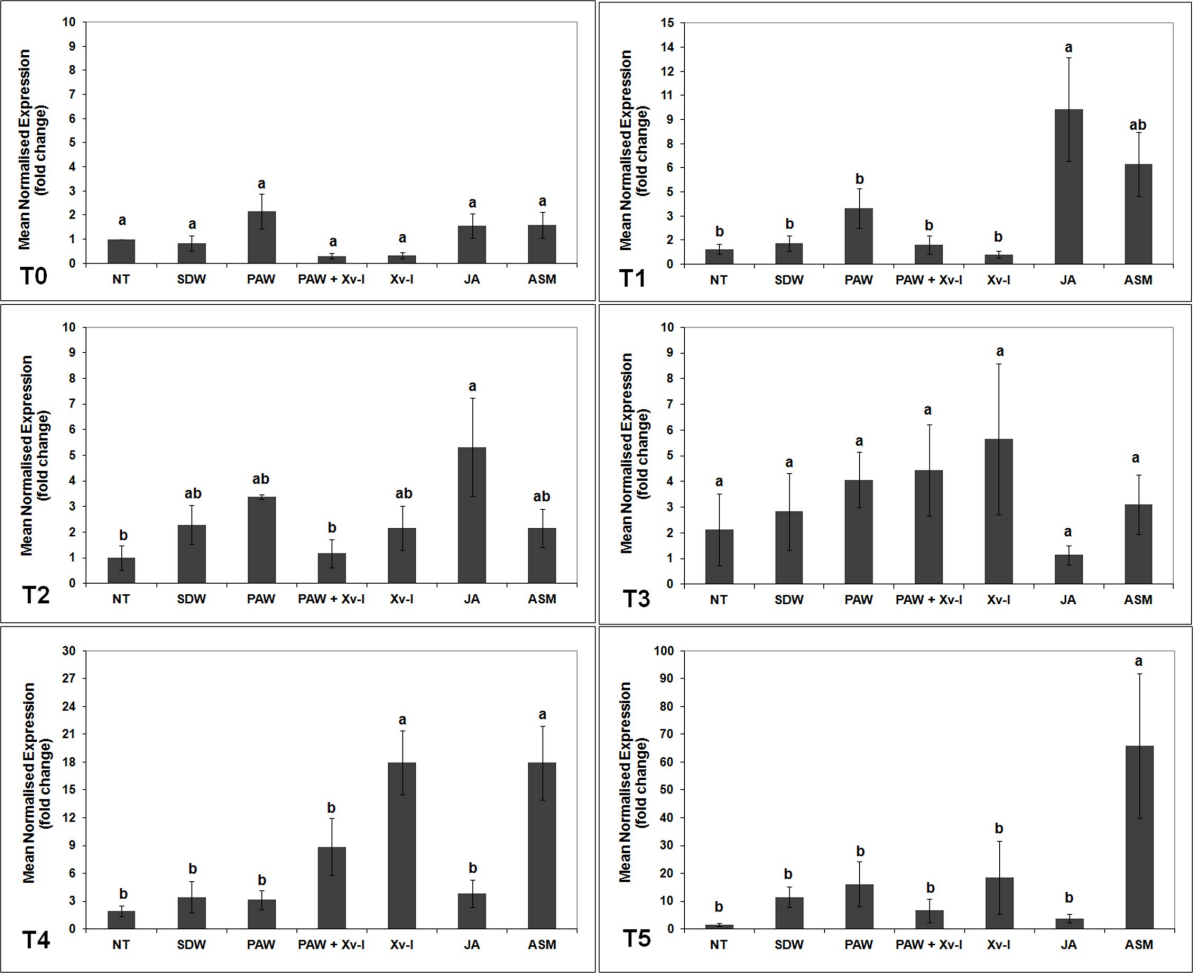
*pr 4* transcription kinetics in tomato leaves. The graph shows the *pr 4* induction at each time point (T0: 1 h; T1: 7 h; T2: 24 h; T3: 48 h; T4: 120 h; T5: 192 h) after treatment/inoculation and the standard error (±SE). Different letters denote significant differences according to the SNK test (p 0.05). NT: non-treated/non-inoculated; SDW: treated plants at root *apparatus* with sterile distilled water; PAW: treated plants at root *apparatus* with plasma activated water; PAW+Xv-I: treated plants at root *apparatus* with plasma activated water and inoculated with *Xanthomonas vesicatoria* sprayed at leaves; Xv-I: inoculated with *Xanthomonas vesicatoria* sprayed at leaves; JA: treated plants at root *apparatus* with jasmonic acid; ASM: treated plants at root *apparatus* with acibenzolar-S-methyl.

**Fig 7:**
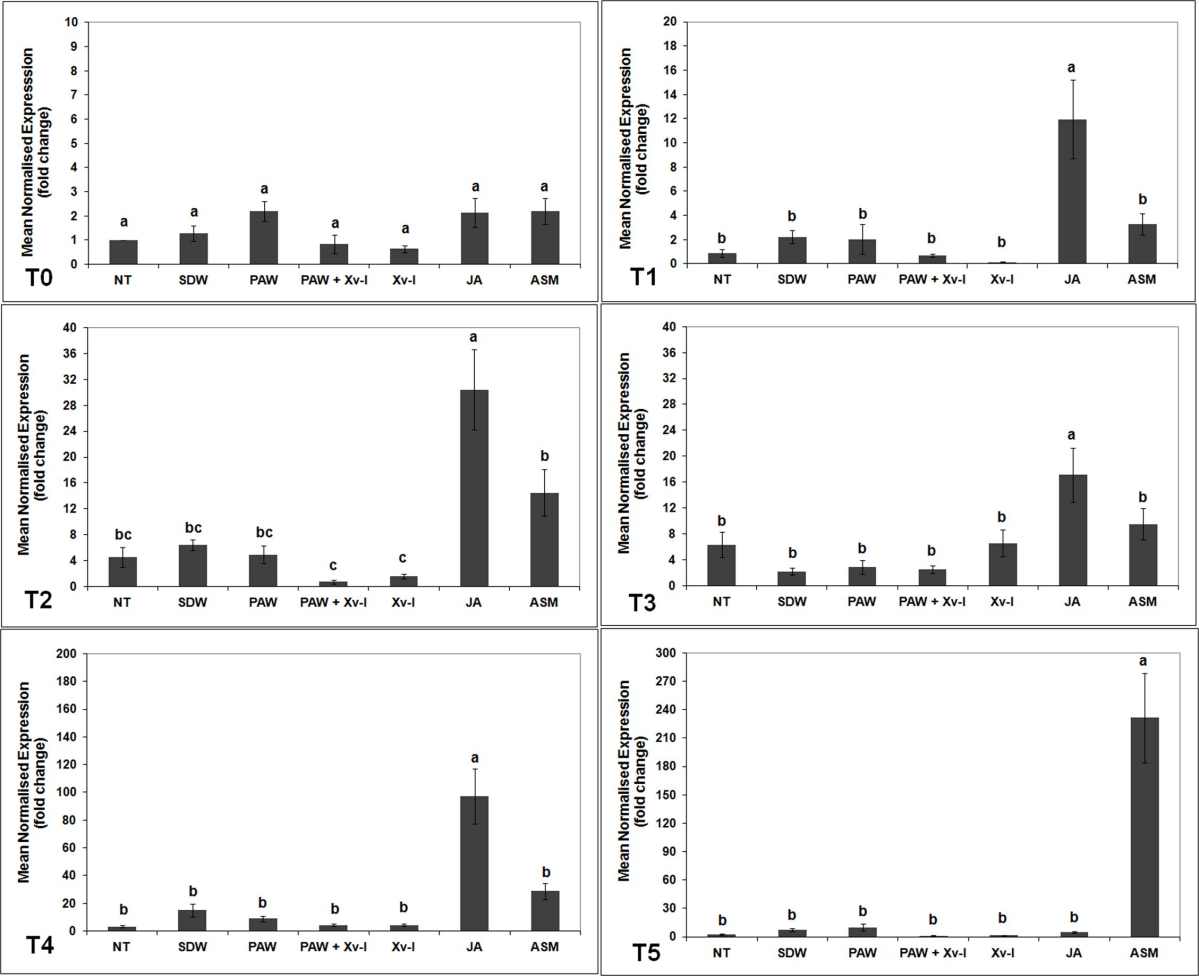
*pr 5* transcription kinetics in tomato leaves. The graph shows the *pr 5* induction at each time point (T0: 1 h; T1: 7 h; T2: 24 h; T3: 48 h; T4: 120 h; T5: 192 h) after treatment/inoculation and the standard error (±SE). Different letters denote significant differences according to the SNK test (p 0.05). NT: non-treated/non-inoculated; SDW: treated plants at root *apparatus* with sterile distilled water; PAW: treated plants at root *apparatus* with plasma activated water; PAW+Xv-I: treated plants at root *apparatus* with plasma activated water and inoculated with *Xanthomonas vesicatoria* sprayed at leaves; Xv-I: inoculated with *Xanthomonas vesicatoria* sprayed at leaves; JA: treated plants at root *apparatus* with jasmonic acid; ASM: treated plants at root *apparatus* with acibenzolar-S-methyl.

The transcription kinetics of *pr1a*, *pr4* and *pr5* genes, encoding post-infection pathogenesis-related proteins (PRs), did not show significant increases in the tomato plants treated with PAW and PAW+Xv-I, when compared to those of NT and SDW treatments, at all time points evaluated (Figs 5–7).

The plants inoculated with the pathogen highlighted a statistical increase of *pr1a* gene transcript quantity after 120 h (T4) (*ca*. 387.0-fold) in comparison to that registered in NT (*ca*. 11.0-fold) and SDW treatments (*ca*. 17.0-fold) (Fig 5). As concerns the plants treated with JA, *pr1a* transcripts abundance significantly increased from T0 (1 h) to T3 (48 h); in this last time point, the transcriptional activity rose at the highest level with respect of all time points (*ca*. 69.8-fold). In the further time points (T4 and T5) such transcription level decreased and it was comparable to that of NT and SDW (Fig 5). The ASM treatment induced a significant increase of *pr1a* gene, which resulted higher than that observed in NT and SDW, in particular, transcript number reached *ca*. 5.3- and 40.0-fold at 1 h and 7 h, respectively; on the other hand, at 120 h (T4) the *pr1a* gene transcription level was *ca*. 402.0-fold, comparable to Xv-I plants (*ca*. 387.0-fold). At 192 h (T5), the number of transcripts in ASM plants was the highest reaching *ca*. 2600.0-fold (Fig 5), while that of Xv-I plants resulted *ca*. 777.0-fold. The increase of *pr1a* in response to pathogen infection suggests the activation of SA pathway, which in known to be related to biotrophic pathogens [22;64].

On the other hand, significant increase of *pr4* gene transcript quantity in Xv-I plants was also found after 120 h (T4) (*ca*. 18.0-fold), this in comparison to that recorded in NT (*ca*. 2.0-fold) and SDW treatments (*ca*. 3.5-fold) (Fig 6); this demonstrates that *pr4* gene is also involved in plant resistance, through the activation of JA/ethylene pathway (basically elicited by necrotrophic pathogens), against Xv, thus confirming its hemibiotrophic lifestyle [22,64–66]. In the JA treatment, an early rise of the *pr4* gene transcripts started at 7 h (*ca*. 9.6-fold), and resulted the highest until 24 h (*ca*. 5.3-fold); this transcription decreased to the baseline until the last time point (192 h, Fig 6). In plants treated with ASM, the *pr4* gene transcript level rose to high levels as well, at 120 h and 192 h it reached *ca*. 18.0- and 65.8-fold, respectively.

As concerns Xv inoculated tomato plants (Xv-I), they did not show rise of *pr5* gene transcription at any time point; in fact, it was similar to that observed in negative controls (Fig 7), thus indicating that *pr5* gene might be not involved in plant resistance against Xv within 192 h after inoculation with the pathogen. On the contrary, in the JA treated plants, *pr5* gene transcription levels started at 7 h (*ca*. 11.9-fold) until the highest level at 120 h (*ca*. 97.2-fold) (Fig 7). Furthermore, ASM induced the highest level of *pr5* gene transcription at 192 h (T5) resulted *ca*. 230.0-fold after treatment application.

In tomato plants inoculated with the pathogen (Xv-I), though the induction of the *pr1a* and *pr4* gene transcript was evaluated, that of *pr5* and *erf* genes, usually involved in both SA and JA/ethylene pathways [22,65], respectively, did not resulted.

## Conclusions

PAW did not show antimicrobial activity against Xv in the *in vitro* experiments using the diffusion and dilution methods.

Nevertheless, in the *in planta* experiments against bacterial leaf spot of tomato, the disease severity resulted lower when PAW was applied 1 and 24 h before the pathogen inoculation, providing RP of 61% and 51%, respectively. When tomato plants, belonging to different cultivars, were treated using PAW six days before the inoculation, the RPs were reduced to *ca*. 38%. In addition, the transcriptomic experiments highlighted the *pal* involvement in response to PAW treatments and against the pathogen; *pal* gene transcription levels resulted significantly high between 1 and 48 h until their decrease, after 192 h from PAW application.

These results indicate that *pal* gene might be a key factor in response to PAW pre-treatments, nonetheless, further investigations are needed to explain the lack of PRs transcript increase. In particular the studies should deeply investigate as concerns the phenylpropanoid pathway, which leads the production of phytoalexins and phenolic substances, or the enhancement of different pathways and to the transcription of defence related genes or transcription factors not investigated in the present study.

## Supporting information

S1 Fig *In vitro* experiment (Diffusion method). The histogram shows the inhibition haloes of *Xanthomonas vesicatoria* growth resulted from *in vitro* assays by using diffusion method.
(TIF)

S2 Fig *In vitro* experiment (Dilution method). The histogram shows the inhibition of *Xanthomonas vesicatoria* population in *in vitro* assays by using broth dilution method.
(TIF)
