# Supplementary figures and images for: Plasma activated water as resistance inducer against bacterial leaf spot of tomato
Source: PLoS One. 2019 May 31;14(5):e0217788. doi: 10.1371/journal.pone.0217788 (PMC6544305; doi:10.1371/journal.pone.0217788)

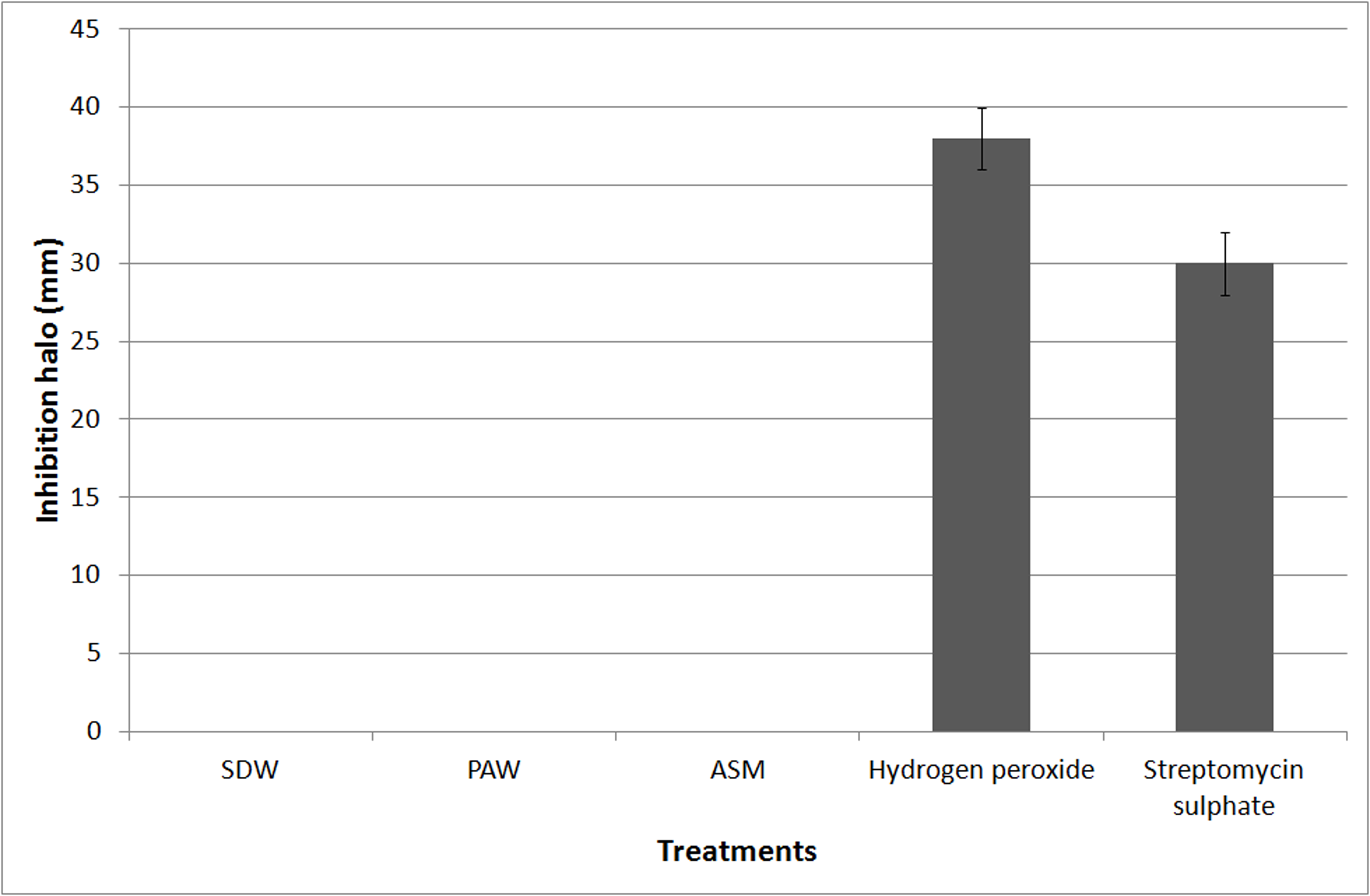

Supplement: S1 Fig — The histogram shows the inhibition haloes of Xanthomonas vesicatoria growth resulted from in vitro assays by using diffusion method. (TIF) [file pone.0217788.s001.tif]

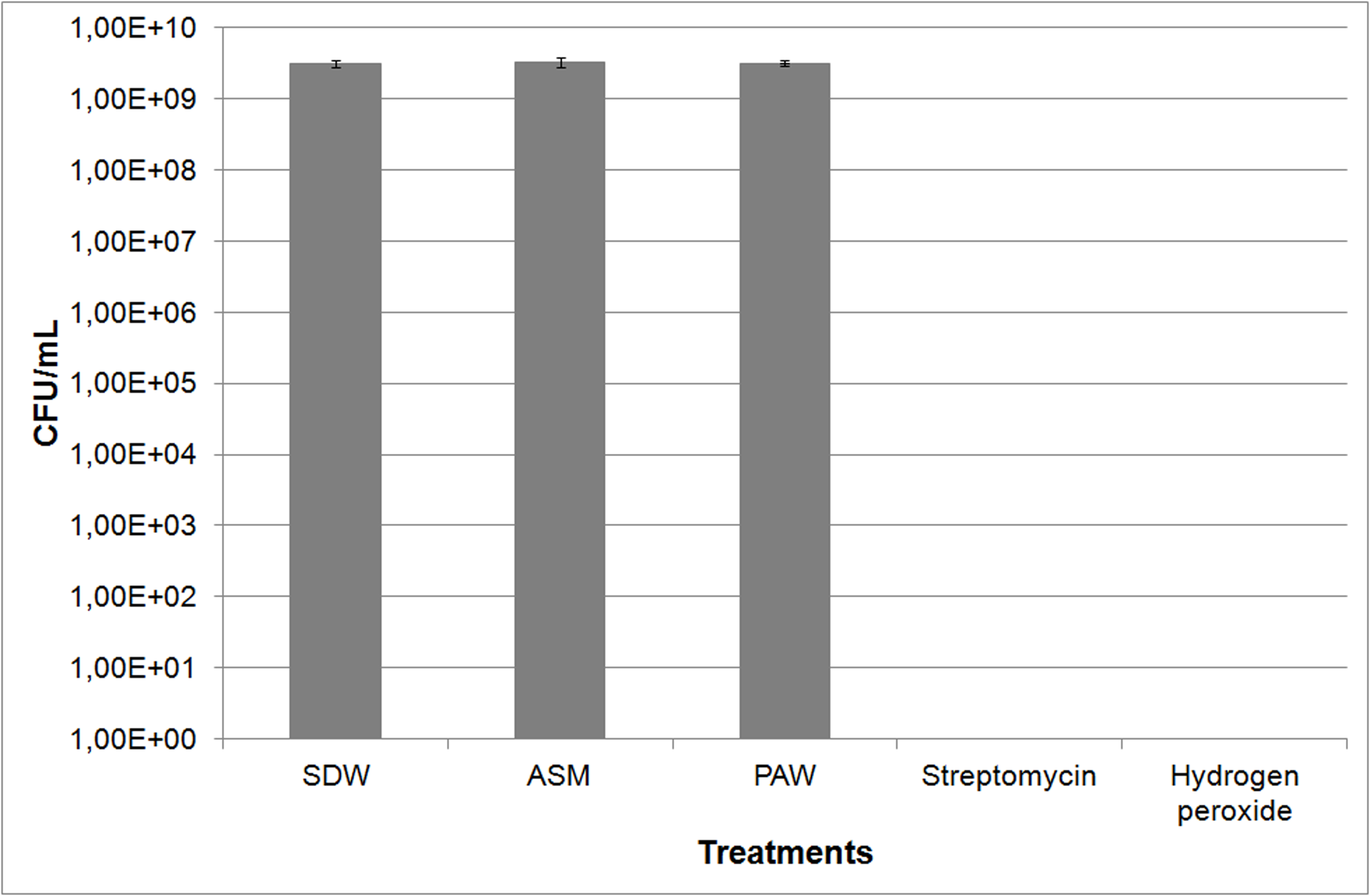

Supplement: S2 Fig — The histogram shows the inhibition of Xanthomonas vesicatoria population in in vitro assays by using broth dilution method. (TIF) [file pone.0217788.s002.tif]
